# Comparative Study of Hirustasin Superfamily Gene Expression in Two Medicinal Leeches, *Hirudinaria manillensis* and *Whitmania pigra*

**DOI:** 10.3390/genes16111332

**Published:** 2025-11-05

**Authors:** Rujiao Sun, Rui Ai, Jingjing Yin, Jianli Cheng, Zuhao Huang, Lizhou Tang, Zichao Liu, Qingqian Zeng, Fang Zhao, Gonghua Lin

**Affiliations:** 1 School of Life Sciences, Key Laboratory of Jiangxi Province for Biological Invasion and Biosecurity, Jinggangshan University, Ji’an 343009, China; sunrujiao2022@163.com (R.S.); 18008109213@163.com (R.A.); m15656142610@163.com (J.Y.); 9920100038@jgsu.edu.cn (J.C.); hzhow@163.com (Z.H.); 2 College of Life Sciences, Jiangxi Normal University, Nanchang 330022, China; tanglizhou@163.com; 3 School of Agronomy and Life Sciences, Kunming University, Kunming 650214, China; abclzc@aliyun.com; 4 Guangdong Institute of Traditional Chinese Medicine, Guangzhou 510655, China; zqqlisa@163.com

**Keywords:** *Hirudinaria manillensis*, *Whitmania pigra*, hirustasin gene superfamily, transcriptomics, dominantly expressed genes, medicinal leeches

## Abstract

Background/Objectives: Leeches constitute a pharmacologically significant animal group in traditional medicine due to their antithrombotic peptides, which include numerous members of the hirustasin gene superfamily. However, a comparative expression profile of this pharmaceutically important family across different leech species is lacking. Methods: This study conducted a comparative transcriptomic analysis of hirustasin gene superfamily expression in the hematophagous leech *Hirudinaria manillensis* and the non-hematophagous leech *Whitmania pigra*. Results: The total expression of the hirustasin gene superfamily, quantified as transcripts per million (TPM), showed no significant difference (*p* = 0.237) between *H. manillensis* (11,802.60 ± 1596.59) and *W. pigra* (8623.12 ± 965.96). However, both species exhibited pronounced intergenic expression heterogeneity. Five dominantly expressed genes (TPM > 1000) in *H. manillensis* and three in *W. pigra* were identified, collectively comprising 81% and 62% of the total hirustasin gene superfamily expression per species, respectively. Critically, the dominantly expressed genes exhibited no phylogenetic correspondence between species. Integrating expression profiles with phylogenetic reconstruction identified five high-potential candidate genes: *poecistasin_Hman2*, *hirustasin_like_Hman01*, *hirustasin_like_Hman11*, *guamerin_Wpig*, and *bdellastasin_Wpig*. Population-level analysis revealed marked population-specific expression patterns in *H. manillensis*, contrasting with minimal inter-population divergence in *W. pigra*. Nevertheless, geographically distinct populations of both species showed significant variation in the expression of their respective dominantly expressed genes. Conclusions: These findings provide a set of high-priority candidate genes and insights into their expression characteristics, serving as a starting point for subsequent functional validation and, when integrated with other screening methods, for future antithrombotic drug discovery.

## 1. Introduction

The incidence and mortality of cardiovascular diseases (CVDs) have continued to increase over the past three decades, mainly due to factors such as hypertension, dietary risks, and hypercholesterolemia [1,2,3]. Thrombosis, one of the major pathogenic mechanisms of CVD, can lead to ischemia, infarction, and organ dysfunction [4]. Although existing antithrombotic drugs are widely used in clinical practice, they are often accompanied by adverse effects such as bleeding and hepatotoxicity [5,6]. Therefore, the development of safe and effective natural antithrombotic agents has become an important research direction.

Medicinal leeches have long been used to prevent and treat blood stasis and thrombotic diseases [7,8,9], and they represent an important group of animal-derived medicinal materials. Among them, *Whitmania pigra* (Whitman, 1884) and *Hirudinaria manillensis* (Lesson, 1842) are the two most common leech species in the Chinese medicinal market. *W. pigra* has been officially recognized as the basic source species of “Shuizhi” in the Pharmacopoeia of the People’s Republic of China (PPRC), whereas *H. manillensis*, despite exhibiting potent anticoagulant activity, has not yet been included [10,11,12,13]. The differences between these two species in feeding habits (hematophagous vs. non-hematophagous) and regulatory legitimacy suggest that their anticoagulant functions may originate from molecular-level variations. In particular, members of the hirustasin gene superfamily, which function as serine protease inhibitors in the salivary glands of leeches, play key roles in anticoagulation. Therefore, a comparative analysis of hirustasin gene expression between these two medicinal leeches may help elucidate the molecular basis of their anticoagulant activity and provide insights for the scientific identification of basic source species and the development of antithrombotic drugs.

Leeches maintain blood flow by secreting various bioactive substances with anticoagulant, fibrinolytic, and anti-inflammatory properties, the types and abundances of which are closely related to their feeding characteristics [14,15]. To date, multiple antithrombotic components have been identified in leeches, such as antistasin, hirudin, guamerin, and hirustasin [15,16]. Hirustasin, first isolated in 1994 from *Hirudo medicinalis* [17], inhibits trypsin, chymotrypsin, tissue kallikrein, and neutrophil-derived cathepsin G. Owing to high sequence/functional homology, proteins including hirustasin [17], guamerin [18], bdellastasin [19], poecistasin [20], piguamerin [21], and uncharacterized hirustasin-like proteins [22] constitute the hirustasin gene superfamily. These proteins typically contain a signal peptide and ten conserved cysteine residues [22]. Primarily co-expressed in leech salivary glands, they synergistically inhibit coagulation and proteolysis, significantly enhancing leech anticoagulant efficacy and underscoring the need for systematic research on this gene superfamily.

To optimize the utilization of medicinal leech resources, it is essential to return to their core attribute as *daodi* medicinal materials—ensuring both quality and therapeutic efficacy. In the traditional Chinese medicine (TCM) system, leeches are regarded as important animal-derived *daodi* medicinal materials due to their remarkable blood-activating, stasis-resolving, anticoagulant, and thrombolytic properties [23]. *Daodi* medicinal materials refer to Chinese medicinal substances cultivated, harvested, and processed under specific natural conditions, ecological environments, and traditional techniques, whose quality and clinical efficacy are generally superior to those of the same species produced in other regions, thus earning high esteem in TCM clinical practice [24,25]. This unique quality advantage, associated with specific geographic origins, underscores the importance of elucidating the intrinsic pharmacological material basis of leeches as *daodi* medicinal materials—particularly the expression characteristics of key antithrombotic components such as the hirustasin gene superfamily. Understanding the potential differences in the expression of these critical bioactive components between leeches of different species (e.g., *W. pigra* vs. *H. manillensis*) or from different production areas is a pivotal step toward safeguarding and enhancing the quality and efficacy of *daodi* medicinal materials, and ultimately provides a key basis for optimizing the development and utilization of medicinal leech resources and for screening efficient antithrombotic drug candidates.

Notably, most antithrombotic proteins exhibit significant interspecific divergence. Although prior studies [22] have delineated hirustasin gene superfamily expression patterns within single species, a systematic cross-species expression comparison remains unreported. To further explore the pharmacological activities and therapeutic potential of *H. manillensis* and *W. pigra*, we integrated RNA-Seq datasets from both medicinal leech species and conducted a systematic cross-species comparison of expression levels within the hirustasin gene superfamily to identify candidate genes with potential functional relevance. It should be emphasized that transcriptomic analyses primarily serve to nominate putative functional candidates by revealing differences at the mRNA level, which do not necessarily correspond to physiological activity or anticoagulant efficacy. Previous studies have shown that the mRNA abundance (e.g., TPM) of certain leech anticoagulant factors exhibits a partial correlation with protein abundance or anticoagulant activity [26], although this correspondence is not absolute. The findings of the present study provide a valuable foundation for subsequent protein-level validation and the development of novel antithrombotic therapeutics.

## 2. Materials and Methods

### 2.1. The RNA Sequencing

Live specimens of *H. manillensis* were collected from four Chinese localities: Liuzhou, Guangxi (24°18′ N, 109°24′ E); Zhanjiang, Guangdong (21°12′ N, 110°24′ E); Honghe, Yunnan (23°23′ N, 103°23′ E); and Ding’an, Hainan (19°46′ N, 110°27′ E). *W. pigra* specimens were obtained from four additional sites: Wuhan (30°33′ N, 114°18′ E) and Zhongxiang (31°07′ N, 112°38′ E) in Hubei Province; Baodi, Tianjin (39°43′ N, 117°30′ E); and Yibin, Sichuan (28°45′ N, 104°36′ E). All individuals were captured alive, with sampling site coordinates recorded via GPS. Sample collection was conducted from May to July 2023, corresponding to the main active season of leeches. The sampling sites were shallow freshwater habitats, including ponds, paddy fields, and irrigation ditches, with ambient water temperatures ranging from 25 °C to 30 °C. All specimens were wild-caught and morphologically identified on-site to confirm species identity. A total of 10 individuals were collected from each geographic population, resulting in 80 samples in total (2 species × 4 populations × 10 individuals). Immediately after collection, the specimens were dissected to remove the digestive tract, and tissue samples were stored at −80 °C to prevent RNA degradation. Total RNA was isolated from cephalic tissues using TRIzol Reagent (Invitrogen, Carlsbad, CA, USA) and purified with the RNeasy Mini Kit (Qiagen, Chatsworth, CA, USA). High-integrity RNA samples were used to construct cDNA libraries (avg. insert size: 350 bp) with Illumina-compatible reagents. RNA sequencing was performed on the BGISeq platform. Raw reads were quality-controlled using fastp v0.20.0 [27], yielding high-quality clean reads for downstream analyses.

### 2.2. Expression Analysis of Hirustasin Gene Superfamily

All coding sequences (CDSs) derived from whole-genome structural annotation served as reference templates. A sequence index was constructed using Salmon [28], to which transcriptomic reads were mapped (k-mer size = 31). Gene expression was quantified as transcripts per million (TPM), representing normalized relative transcript abundance. The cumulative TPM values of all hirustasin gene superfamily members per sample were summed to represent total superfamily gene expression.

This study compared total hirustasin gene superfamily gene expression between *H. manillensis* and *W. pigra* using the Mann–Whitney U test. To validate statistical method appropriateness, data normality was first assessed via the Shapiro–Wilk test. Given confirmed non-normality, the non-parametric Mann–Whitney U test was subsequently applied for comparative analysis of non-normally distributed data. This methodological choice guaranteed statistical rigor and analytical reliability. Data visualization was performed in RStudio v2024.12.1 [29] using R v4.4.2 [30] and ggplot2 v3.5.2 [31].

For the relative expression analysis of transcriptome data, statistical analyses were conducted using SPSS 26.0 (IBM Corp., Armonk, NY, USA) [32]. The mean ± SE values were calculated for each member of the hirustasin gene superfamily in *Hirudinaria manillensis* and *Whitmania pigra*. Pie charts were produced in Origin 2021 (OriginLab Corporation, Northampton, MA, USA) [33] to illustrate the proportion of dominantly expressed genes (TPM > 1000) relative to the total expression of the gene family in each species. Eight dominantly expressed genes with TPM > 1000 were subsequently selected for comparison. The Shapiro–Wilk test revealed that the data did not meet the assumptions of normality and homogeneity of variance; therefore, non-parametric methods were applied for statistical analysis. The Kruskal–Wallis test was used to evaluate significant differences in gene expression among the eight dominant genes. Normalized TPM data were visualized as bar plots using GraphPad Prism 10.1.2 (GraphPad Software, San Diego, CA, USA) [34], revealing differential gene expression patterns of hirustasin gene superfamily members.

### 2.3. Phylogenetic Analysis of the Hirustasin Gene Superfamily in H. manillensis and W. pigra

Amino acid sequences of the hirustasin gene superfamily from *H. manillensis* and *W. pigra* were collected and aligned using MEGA X v10.2 [35] with the ClustalW algorithm under default parameters. The resulting multiple sequence alignment was saved in FASTA format and used as the input for phylogenetic analysis in IQ-TREE v2.2.0 [36]. Phylogenetic trees were constructed using the Maximum Likelihood method, with the best-fit substitution model automatically selected by the ModelFinder module based on the Bayesian Information Criterion. Branch support was assessed using 1000 ultrafast bootstrap replicates. The resulting phylogenetic tree was visualized using FigTree v1.4.4 [37], and graphical refinement and annotation were performed with Inkscape v1.2.2 [38]. Based on the phylogenetic tree, the hirustasin gene superfamily members from *H. manillensis* and *W. pigra* were classified into nine monophyletic clades, designated B1 through B9. Interspecies differences in gene expression within each clade were evaluated using the Mann–Whitney U test.

### 2.4. Analysis of Expression Between Populations

The gene expression of hirustasin gene superfamily members from *H. manillensis* and *W. pigra* was combined for population-level analysis. Using SPSS 26.0 (IBM Corp., Armonk, NY, USA) [32], the mean ± standard error (Mean ± SE) of each gene was calculated for different populations within each species. The Shapiro–Wilk test was then performed, and the results indicated that the data did not meet the assumptions of normality and homogeneity of variance. Therefore, non-parametric statistical methods were deemed appropriate for subsequent analysis. The Kruskal–Wallis test was applied via the “Analyze” → “Nonparametric Tests” → “Independent Samples” module to evaluate whether significant differences existed in the total gene expression of the hirustasin gene superfamily among populations within each species, as well as in the gene expression of dominant genes across populations in both species. Finally, a hierarchical clustering heatmap was generated using TBtools-II (Toolbox for Biologists) v2.327 [39]. Row normalization was performed using Z-score transformation, and the clustering algorithm was based on Euclidean distance with average linkage.

## 3. Results

### 3.1. Comparison of Expression Between Species

**Figure 1:**
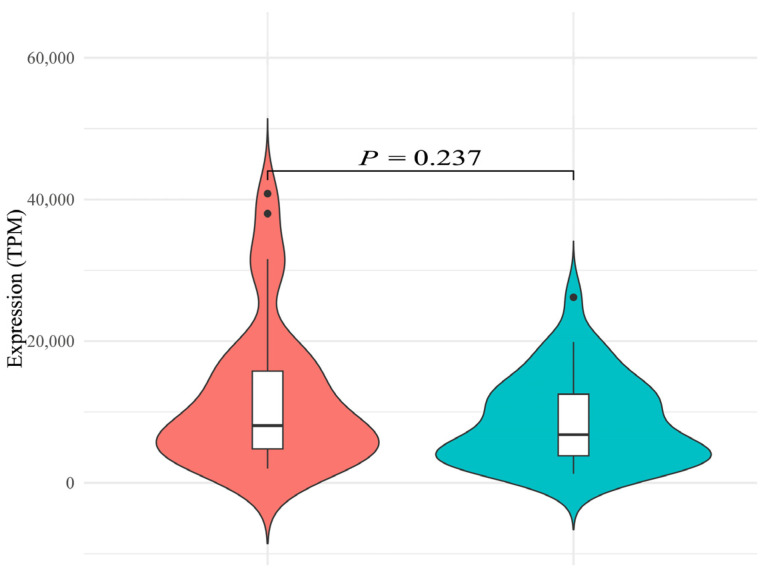
Violin plot with overlaid boxplot showing the total gene expression of the hirustasin gene superfamily in *Hirudinaria manillensis* (**left**) and *Whitmania pigra* (**right**). The violin plot outlines represent the kernel density distribution of expression values; greater widths indicate a higher density of samples at that gene expression. Overlaid white boxplots indicate the median (bold horizontal line within the box), interquartile range (box boundaries), and whiskers extending to 1.5 × IQR beyond the upper and lower quartiles. Dots outside the whiskers denote outliers. The statistical annotation above the violin plots (*p* = 0.237, Mann–Whitney U test) indicates that there is no significant difference in total hirustasin gene expression between the two species.

To compare the overall expression differences of the hirustasin gene superfamily between the two species, we first analyzed the total gene expression. The total gene expression (TPM) of the hirustasin gene superfamily in *H. manillensis* was 11,802.60 ± 1596.59, while in *W. pigra*, it was 8623.12 ± 965.96 (Supplementary Tables S1 and S2). Shapiro–Wilk tests revealed that the data from both *H. manillensis* (*p* = 0.000) and *W. pigra* (*p* = 0.004) significantly deviate from normal distribution. Therefore, the non-parametric Mann–Whitney U test was used for statistical comparison. The result showed no significant difference between the two species (*Z* = −1.184, *p* = 0.237), suggesting that the total gene expression of the hirustasin gene superfamily were not statistically different between *H. manillensis* and *W. pigra*. As shown in Figure 1, the gene expression in *H. manillensis* displayed considerable inter-sample variability, with a wider violin plot contour and extended tails at both high and low gene expression, indicating a dispersed distribution and potentially greater biological variability. In contrast, *W. pigra* exhibited a narrower contour, reflecting a more concentrated expression distribution across samples, which may suggest tighter regulatory control of hirustasin gene expression in this species.

**Figure 2:**
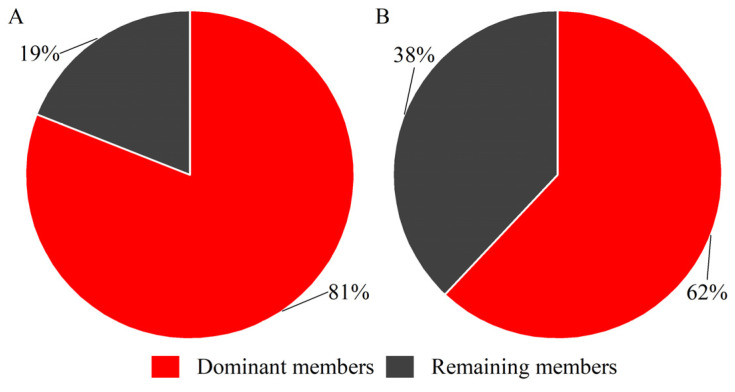
Pie charts showing the proportional expression of dominant members of the hirustasin gene superfamily in *H. manillensis* and *W. pigra* ((**A**), *H. manillensis*; (**B**), *W. pigra*).

Comparative analysis of individual gene expression revealed highly skewed distributions within the hirustasin gene superfamily of both *H. manillensis* and *W. pigra*, despite comparable total expression (Table 1). Genes with TPM > 1000 were classified as dominantly expressed genes, yielding five such genes in *H. manillensis* (*hirustasin_like_Hman01*, *hirustasin_like_Hman04*, *hirustasin_like_Hman11*; *guamerin_Hman*; *poecistasin_Hman2*) and three in *W. pigra* (*hirustasin_like_Wpig5*; *guamerin_Wpig*; *bdellastasin_Wpig*). The homologs of these dominantly expressed genes have been confirmed in previous studies to participate in key hemostatic regulatory processes. Both *hirustasin_like_Hman01* and *hirustasin_like_Hman04* contain a typical Kunitz-type serine protease inhibitor domain and show high sequence similarity to the reported Hirustasin protein, suggesting that they may inhibit tissue kallikrein and neutrophil proteases, thereby contributing to anti-inflammatory and immune-regulatory functions [17]. *Hirustasin_like_Hman11* shares high similarity with the characterized *poeciguamerin* sequence, which exhibits both anticoagulant and anti-inflammatory activities, indicating that this gene may possess dual functionality [40]. *Hirustasin_like_Wpig5*, *guamerin_Wpig*, and *guamerin_Hman* show strong homology to Guamerin-like proteins, which have been identified as inhibitors of coagulation factor Xa (FXa); thus, these genes are likely to play major roles in anticoagulation by blocking FXa activity [15,18]. The homolog of *poecistasin_Hman2* has been reported to inhibit platelet aggregation, suggesting a potential involvement in antiplatelet mechanisms [20]. The homolog of *bdellastasin_Wpig* is known to specifically inhibit thrombin, implying that it may directly interfere with the terminal step of the coagulation cascade [19]. These dominant genes accounted for a substantial proportion of the total expression—representing 81% in *H. manillensis* and 62% in *W. pigra* (Figure 2)—further underscoring their significance as reference candidates for anticoagulant gene screening.

**Figure 3:**
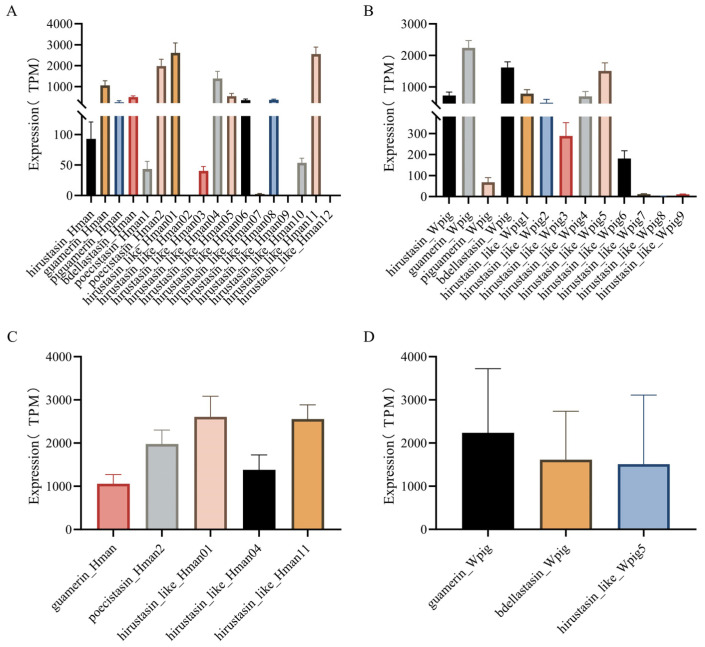
Bar plots showing gene expression of hirustasin gene superfamily members. (**A**) Gene expression of hirustasin superfamily genes in *H. manillensis*. (**B**) Gene expression of hirustasin superfamily genes in *W. pigra*. (**C**) Dominantly expressed hirustasin genes (TPM > 1000) in *H. manillensis*. (**D**) Dominantly expressed hirustasin genes (TPM > 1000) in *W. pigra*.

Subsequently, the eight dominantly expressed genes—*guamerin_Hman*, *poecistasin_Hman2*, *hirustasin_like_Hman01*, *hirustasin_like_Hman04*, *hirustasin_like_Hman11*, *guamerin_Wpig*, *bdellastasin_Wpig*, and *hirustasin_like_Wpig5*—were subjected to Kruskal–Wallis analysis. The results revealed highly significant differences among these genes (*H* = 37.407, *df* = 7, *p* < 0.001). Pairwise comparisons indicated that *guamerin_Hman* significantly differed from *hirustasin_like_Hman01*, *hirustasin_like_Hman11*, and *guamerin_Wpig*, while *hirustasin_like_Hman04* also showed significant differences from *hirustasin_like_Hman11* and *guamerin_Wpig*. These findings suggest that these genes are of particular interest and warrant further investigation (Figure 3).

### 3.2. Phylogenetic Relationships of Hirustasin Gene Superfamily in H. manillensis and W. pigra

**Figure 4:**
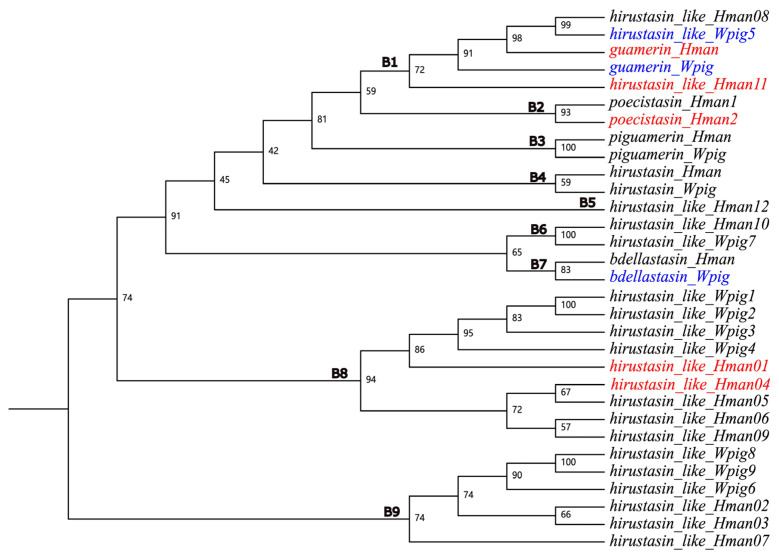
Phylogenetic relationships among hirustasin gene superfamily members from *H. manillensis* and *W. pigra*. Red branches represent dominantly expressed genes in *H. manillensis*, while blue branches represent dominantly expressed genes in *W. pigra*.

To assess phylogenetic correspondence of the dominantly expressed genes, we constructed a hirustasin gene superfamily phylogeny (Figure 4 and Supplementary Data S1). The tree revealed intermingled clustering of *H. manillensis* and *W. pigra* sequences across clades. Crucially, most dominantly expressed genes lacked phylogenetically conserved orthology (Table 2), with no evidence of co-dominant expression in closely related ortholog pairs. Mann–Whitney U tests further demonstrated significant interspecific expression differences in all clades except B8 (where expression was comparable). Collectively, these results indicate that *H. manillensis* and *W. pigra* exhibit species-specific expression patterns within the hirustasin gene superfamily.

Subsequently, for each monophyletic clade exhibiting dominant gene expression in at least one species, we selected one representative gene per species. With the exception of B1, the gene expression of representative genes from the remaining clades showed substantial differences between *H. manillensis* and *W. pigra*. We further screened for genes with TPM values greater than 1000 and found overlap with previously screened dominantly expressed genes, including *poecistasin_Hman2*, *hirustasin_like_Hman01*, *hirustasin_like_Hman11*, *guamerin_Wpig*, and *bdellastasin_Wpig* (Table 3). Moreover, we found that the gene expression of representative genes from monophyletic clades in *H. manillensis* was generally higher than that in *W. pigra*, suggesting a potential correlation between blood-feeding behavior and the upregulation of anticoagulant genes. This pattern also implies a link between gene expression, gene function, and evolutionary relationships.

### 3.3. Comparison of Expression Between Populations

**Figure 5:**
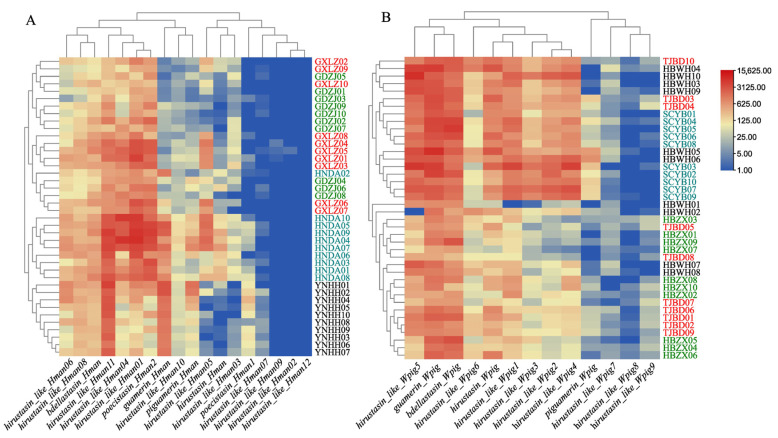
Hierarchical clustering heatmaps based on the gene expression of hirustasin gene superfamily members across geographic populations of two leech species ((**A**), *H. manillensis*; (**B**), *W. pigra*). Rows represent geographic populations, and columns represent hirustasin gene superfamily members. Color gradients indicate gene expression, with red representing high gene expression and blue representing low gene expression. The dendrograms reflect the similarity in gene expression patterns among populations.

To compare the population gene expression of the hirustasin gene superfamily in *H. manillensis* and *W. pigra*, Kruskal–Wallis tests were performed. The results revealed significant differences in total gene expression among geographic populations for both species. In *H. manillensis*, the difference was highly significant (*H* = 19.055, *df* = 3, *p* < 0.001), and in *W. pigra* as well (*H* = 17.501, *df* = 3, *p* = 0.001). However, hierarchical clustering heatmaps demonstrated contrasting inter-population patterns (Figure 5). The clustering of *H. manillensis* populations was more distinct and aligned with geographic origins, whereas *W. pigra* populations exhibited less defined clustering. Notably, the Ding’an, Hainan *H. manillensis* population displayed significantly higher average gene expression than other populations (Table 4). This suggests region-specific *H. manillensis* populations may possess enhanced pharmacological potential, underscoring the importance of *daodi* medicinal properties—where specific geographic origins confer superior therapeutic quality in traditional medicine. These findings provide critical insights for evaluating regional authenticity and ethnopharmacological value of *H. manillensis*.

We further observed that all previously identified dominant genes, except *bdellastasin_Wpig* (*H* = 7.169, *df* = 3, *p* = 0.067), exhibited significant expression differences among geographic populations within their species. Specifically, *hirustasin_like_Hman01* (*H* = 24.745, *df* = 3, *p* < 0.001), *hirustasin_like_Hman11* (*H* = 25.073, *df* = 3, *p* < 0.001), and *poecistasin_Hman2* (*H* = 19.889, *df* = 3, *p* < 0.001) in *H. manillensis*, along with *guamerin_Wpig* (*H* = 14.033, *df* = 3, *p* = 0.003) in *W. pigra*, showed significant inter-population expression variation (Table 4 and Table 5). These five genes may thus serve as key molecular markers for *daodi* properties in leech-derived materials, likely driving regional variations in pharmacological efficacy observed in traditional medicines.

## 4. Discussion

Cardiovascular disease remains the leading cause of death globally [2], and currently available antithrombotic drugs are often associated with adverse effects such as bleeding and hepatotoxicity [6]. Therefore, the development of more effective and safer therapeutic agents has become an urgent priority. This study focused on the hirustasin gene superfamily in two medicinal leech species—*H. manillensis*, a traditional hematophagous species, and *W. pigra*, a non-hematophagous species listed in the Pharmacopoeia of the People’s Republic of China. Through multidimensional expression analyses, we screened key pharmacologically relevant genes, providing a molecular basis for the discovery and development of novel antithrombotic agents.

### 4.1. Legitimacy of the Basic Sources of H. manillensis and W. pigra as Medicinal Leeches

The legitimacy of a medicinal material’s basic source refers to the extent to which its source species is officially recognized by national or regional pharmacopoeias, supported by historical documentation, taxonomic accuracy, pharmacological validation, and compliance with safety standards. *H. manillensis* and *W. pigra* are the two most common leech species in the Chinese medicinal market, valued for their rapid growth and large body size. However, their medicinal attributes and regulatory status differ markedly. Owing to its hematophagous behavior, *H. manillensis* is generally considered by the scientific and medical communities to be a superior source of antithrombotic compounds and is presumed to possess greater therapeutic potential, arguably fulfilling ethnopharmacological and scientific criteria for legitimacy. Yet, despite its documented historical usage and modern pharmacological evidence, it has not been included in the Pharmacopoeia of the PPRC [11], and its legal recognition is currently confined to provincial drug standards in regions such as Yunnan and Guangxi [41,42]. From a national regulatory perspective, it remains outside the category of statutory medicinal materials, imposing additional approval requirements and costs for pharmaceutical development. In contrast, *W. pigra*, although non-hematophagous and feeding mainly on mollusks, meets the historical and morphological definitions of “Mazhi” in classical materia medica, has been listed in the PPRC since 1963, and is fully recognized at the national level as a legitimate basic source species of medicinal leeches [10,43,44]. Importantly, our recent studies have repeatedly confirmed that *W. pigra* retains substantial antithrombotic activity [45,46], further supporting its continued legal inclusion as a pharmacopeial basic source species. This dichotomy illustrates that while both species possess substantial ethnopharmacological and scientific legitimacy as basic sources, only *W. pigra* currently enjoys complete legal legitimacy under national standards, whereas *H. manillensis* remains legally limited despite its pharmacological merits. Given the growing body of molecular, pharmacological, and clinical evidence, we recommend that *H. manillensis* be considered for inclusion in the PPRC to standardize its application and facilitate its safe, effective use in modern medicine.

Our findings indicate that, despite the pronounced dietary divergence between the two species, the overall gene expression of the hirustasin gene superfamily does not differ significantly. We speculate that the ancestors of *W. pigra* possessed anticoagulant capacities comparable to those of medicinal leeches, and that the relatively recent shift in feeding habits has not yet led to a comprehensive down-regulation or loss of antithrombotic genes. However, high gene expression does not invariably correspond to high protein yield or potent bioactivity. For instance, although the genome of *W. pigra* contains seven hirudin genes compared to only five in *H. manillensis*, previous studies have demonstrated that three of the five hirudins in *H. manillensis* exhibit pronounced anticoagulant activity, whereas only one of the seven in *W. pigra* displays comparable anticoagulant activity [22,47]. Although no significant difference was observed in the overall gene expression of the hirustasin gene superfamily between *H. manillensis* and *W. pigra*, it is premature to conclude comparable anticoagulant efficacy based solely on gene expression data. Subsequent validation through recombinant protein expression, functional assays, and related experiments is essential. These investigations will establish a robust scientific foundation for the rational selection of raw materials and facilitate the development of effective antithrombotic therapeutics.

### 4.2. Daodi Medicinal Properties of Medicinal Leeches

From a pharmaceutical perspective, the stability of the geographical source of medicinal materials is also a key determinant of both therapeutic efficacy and safety. Inconsistent raw material quality can result in unpredictable pharmacological effects—either subtherapeutic outcomes due to insufficient dosing or adverse reactions from overdosing. Our findings reveal that although significant differences exist among regional populations of both *H. manillensis* and *W. pigra*, the expression patterns of the hirustasin gene superfamily in *H. manillensis* exhibit strong geographic cohesion. Individuals from the same populations (e.g., YNHH and HNDA) tend to cluster together based on gene expression patterns. In contrast, *W. pigra* populations from different regions show weak clustering, indicating a lower degree of regional differentiation. These observations suggest that the two species should be treated differently with respect to raw material stability and *daodi* medicinal properties. For *W. pigra*, geographic origin may not be a critical factor in evaluating its medicinal value. However, for *H. manillensis*, regional origin should be considered an essential variable [45]. Future research should conduct more detailed experimental studies on inter-population differences in *H. manillensis*, including gene expression analysis, recombinant protein production, and functional assays, to identify populations with the strongest anticoagulant potential. Ultimately, the region with the most bioactive population should be designated as the core production area for *H. manillensis*, thereby laying the foundation for standardized variety development.

The well-known principle that “high-quality medicine comes from high-quality medicinal materials” reflects a fundamental truth in traditional medicine. With the increasing industrialization and commercialization of traditional Chinese medicine (TCM), concerns surrounding the quality and consistency of medicinal materials and decoction pieces have become more pronounced. Many people erroneously attribute the perceived instability in TCM efficacy to deficiencies in TCM theory itself. In reality, the instability should be attributed to the poor quality of medicinal materials (specifically, issues with their geographical origin), not to flaws in traditional Chinese medicine. The quality of medicinal materials is not only closely related to species identity but also heavily influenced by their geographic origin, which determines the biosynthesis and accumulation of active constituents in medicinal plants and animals. In China, many commonly used TCMs are distributed across broad geographic ranges. However, variations in topography, soil type, climate, water quality, and ecosystem conditions across eastern, western, southern, northern, and central regions often lead to significant differences in the quality of the same medicinal species. In some cases, these differences can be substantial. For example, the content of puerarin in *Puerariae lobatae* Radix can vary from 0.55% to 7.78% depending on the production region—a more than 14-fold difference [48]. Similarly, the antimalarial compound artemisinin in *Artemisia annua* shows dramatic geographic variation in its content across China, from Hainan Island in the south to Heilongjiang in the north [49]. In *Morindae officinalis* Radix, the accumulation of bioactive compounds is significantly influenced by ecological factors such as geographic environment, temperature, humidity, and soil conditions, leading to considerable differences in quality across production areas [50]. Such variability in medicinal material quality inevitably translates into inconsistent clinical outcomes. There is growing recognition both domestically and internationally that the stability and *daodi* origin of medicinal materials are equally as important as the drug itself in determining efficacy. In response, the National Medical Products Administration of China has issued the Good Agricultural Practices (GAP) for Medicinal Materials, which emphasizes the establishment of production bases in ecologically optimal regions for high-quality medicinal cultivation. In this context, our study provides essential technical evidence to support the scientific evaluation of *daodi* properties in medicinal leeches, offering a foundation for improving consistency, efficacy, and the traceability of TCM raw materials.

### 4.3. Dominantly Expressed Genes

In drug development, cost and efficiency are equally critical considerations. Numerous antithrombotic compounds have been identified in leeches [15,16], and most antithrombotic proteins are derived from multigene families [51], whose members often exhibit high sequence and functional similarity. Given the size and redundancy of these families, it would be prohibitively time- and resource-intensive to conduct comprehensive biochemical and functional characterization of every gene member. Moreover, indiscriminate targeting of entire gene families may result in off-target effects and unintended physiological disturbances due to the simultaneous modulation of structurally similar isoforms [52]. To date, 13 members of the hirustasin gene superfamily have been identified in the *W. pigra* genome [47], and 18 members in the *H. manillensis* genome [22]. In this context, prioritizing dominantly expressed genes with distinct evolutionary characteristics based on gene expression and phylogenetic relationships provides a rational and efficient strategy to improve the feasibility and success of downstream experimental validation. This strategy—“from broad to focused, from many to few”—is essential for the effective development of therapeutics targeting multigene families.

Phylogenetic analysis in this study revealed substantial overlap between *H. manillensis* and *W. pigra* in the hirustasin gene superfamily. Most gene clades—such as monophyletic groups B1, B2, B3, B4, B6, and B8—showed mixed distributions between the two species. This pattern may reflect the retention of ancestral genes; prior to speciation, both species likely inherited functionally conserved hirustasin genes whose core domains have maintained their ability to target coagulation factors throughout evolution. The high evolutionary conservation indicates the functional significance of these genes [53], suggesting that those exhibiting interspecific convergence may play critical biological roles. In general, genes with higher gene expression are presumed to contribute more substantially to the pharmacological activity of medicinal resources. Through multi-level comparative analyses of gene expression, we comprehensively examined interspecific differences in the hirustasin gene superfamily between *H. manillensis* and *W. pigra*. Ultimately, we screened five dominantly expressed genes (*hirustasin_like_Hman01*, *hirustasin_like_Hman11*, *guamerin_Wpig*, *poecistasin_Hman2*, and *bdellastasin_Wpig*) as the most promising candidates with potential medicinal value.

Among the five candidate genes screened as having the highest medicinal potential, *poecistasin* from *H. manillensis* has been previously confirmed to possess biological activity and was thus selected as a key gene for experimental investigation [20]. Additionally, *hirustasin_like_Hman11* shares high sequence similarity with the previously reported *poeciguamerin* [40]. The latter has demonstrated dual analgesic and antithrombotic activity [40], suggesting that *hirustasin_like_Hman11* may also represent a highly valuable candidate gene. *Hirustasin_like_Hman01* is the most abundantly expressed gene within the hirustasin gene superfamily of *H. manillensis*, and it is located in the core phylogenetic branch (clade B8). However, this gene has never been reported before, and its functional properties remain unknown. Therefore, elucidating the reason behind the unusually high gene expression of *hirustasin_like_Hman01* and determining whether it possesses bioactivity will be the primary focus of our subsequent investigations. We plan to perform recombinant protein expression and conduct comprehensive functional experiments to verify its biological activity. If potent bioactivity can be demonstrated, this gene may hold considerable development potential and could even serve as a key biomarker for evaluating the medicinal quality of *H. manillensis*. The *guamerin* gene has been repeatedly validated for its analgesic and anti-inflammatory effects in *Hirudo nipponia* (Korean medicinal leech), leading us to speculate that it may exhibit similar activities in *W. pigra* [15]. *Bdellastasin* was first reported in the European medicinal leech *Hirudo medicinalis* [19,54], but has received little attention in subsequent studies. Interestingly, our phylogenetic analysis revealed that *bdellastasin* sequences are highly conserved, consistently forming a distinct monophyletic group across species. This suggests that *bdellastasin_Wpig* may also retain conserved functions and holds considerable pharmacological value.

## 5. Conclusions

In this study, we systematically compared integrated RNA-Seq data of the hirustasin gene superfamily in two medicinal leech species, *H. manillensis* and *W. pigra*, yielding key insights: First, no significant difference was observed in overall hirustasin gene superfamily expression between species (*p* = 0.237), indicating both possess substantial medicinal potential. Although *H. manillensis* remains unlisted in the Pharmacopoeia of the People’s Republic of China (PPRC), its pharmacological value warrants serious consideration. Second, pronounced intra-species gene expression variation exists, with dominant genes accounting for 81% (*H. manillensis*) and 62% (*W. pigra*) of total expression. This highly unbalanced expression pattern lacks clear phylogenetic conservation. Integrating expression levels and evolutionary positions, we identified five genes with maximal therapeutic promise: *poecistasin_Hman2*, *hirustasin_like_Hman01*, *hirustasin_like_Hman11*, *guamerin_Wpig*, and *bdellastasin_Wpig*—prioritizing these for protein activity validation. Third, dominant genes exhibited significant expression divergence across geographic populations in both species. *H. manillensis* demonstrated stronger population clustering, suggesting greater region-specific expression traits. These findings underscore the critical importance of *daodi* medicinal properties in future leech-derived medicinal resource development. Collectively, this study systematically compared the hirustasin gene superfamily between *H. manillensis* and *W. pigra*, identified core candidate genes with potential functional significance, and revealed species- and population-specific expression patterns. These findings provide molecular insights into the *daodi* characteristics of medicinal leeches. Although transcriptomic results do not directly represent physiological activity, this work establishes a molecular framework for exploring the anticoagulant mechanisms of medicinal leeches and offers valuable genetic resources for antithrombotic drug discovery. In the future, integrating expression-based prioritization strategies with structural prediction, molecular docking, computer-aided drug design, and machine learning approaches may help construct a more efficient drug discovery pipeline, thereby supporting the optimized utilization of leech-derived medicinal resources and reducing subsequent development costs.

## Figures and Tables

**Table 1:** TPM values of hirustasin gene superfamily members in *Hirudinaria manillensis* and *Whitmania pigra* (genes with TPM > 1000 are shown in bold).

Species	Genes	TPM (Mean ± SE)
*H. manillensis*	*hirustasin_Hman*	92.72 ± 27.61
	*guamerin_Hman*	**1059.16 ± 213.26**
	*piguamerin_Hman*	242.05 ± 75.62
	*bdellastasin_Hman*	498.73 ± 50.99
	*poecistasin_Hman1*	43.12 ± 12.74
	*poecistasin_Hman2*	**1979.36 ± 323.89**
	*hirustasin_like_Hman01*	**2608.04 ± 476.68**
	*hirustasin_like_Hman02*	0.05 ± 0.05
	*hirustasin_like_Hman03*	40.40 ± 6.97
	*hirustasin_like_Hman04*	**1377.83 ± 346.05**
	*hirustasin_like_Hman05*	539.67 ± 128.56
	*hirustasin_like_Hman06*	350.02 ± 52.86
	*hirustasin_like_Hman07*	2.53 ± 0.83
	*hirustasin_like_Hman08*	360.13 ± 31.59
	*hirustasin_like_Hman09*	0.10 ± 0.07
	*hirustasin_like_Hman10*	53.60 ± 7.49
	*hirustasin_like_Hman11*	**2555.10 ± 330.36**
	*hirustasin_like_Hman12*	0.00 ± 0.00
*W. pigra*	*hirustasin_Wpig*	725.32 ± 109.63
	*guamerin_Wpig*	**2237.09 ± 234.68**
	*piguamerin_Wpig*	68.07 ± 22.53
	*bdellastasin_Wpig*	**1615.72 ± 176.90**
	*hirustasin_like_Wpig1*	782.95 ± 128.68
	*hirustasin_like_Wpig2*	495.12 ± 103.76
	*hirustasin_like_Wpig3*	288.65 ± 63.01
	*hirustasin_like_Wpig4*	697.36 ± 152.85
	*hirustasin_like_Wpig5*	**1507.97 ± 253.54**
	*hirustasin_like_Wpig6*	181.25 ± 36.50
	*hirustasin_like_Wpig7*	12.01 ± 1.87
	*hirustasin_like_Wpig8*	1.34 ± 0.37
	*hirustasin_like_Wpig9*	10.27 ± 2.21

TPM, transcripts per million; SE, standard error.

**Table 2:** Comparison of average gene TPMs between monophyletic clades of *H. manillensis* and *W. pigra* (genes with clade-average TPM > 1000 are shown in bold).

Branch	Monophyletic Group		TPM (Mean ± SE)		Mann–Whitney U Test
	*H. manillensis*	*W. pigra*	*H. manillensis*	*W. pigra*	*p*
B1	*guamerin_Hman* *hirustasin_like_Hman08* *hirustasin_like_Hman11*	*guamerin_Wpig* *hirustasin_like_Wpig5*	**1324.80 ± 155.06**	**1872.53 ± 176.47**	0.000
B2	*poecistasin_Hman1* *poecistasin_Hman2*	*—*	**1011.24 ± 194.92**	—	—
B3	*piguamerin_Hman*	*piguamerin_Wpig*	242.05 ± 75.62	68.07 ± 22.53	0.002
B4	*hirustasin_Hman*	*hirustasin_Wpig*	92.72 ± 27.61	725.32 ± 109.63	0.000
B5	*hirustasin_like_Hman12*	*—*	0.00 ± 0.00	—	—
B6	*hirustasin_like_Hman10*	*hirustasin_like_Wpig7*	53.60 ± 7.49	12.01 ± 1.87	0.000
B7	*bdellastasin_Hman*	*bdellastasin_Wpig*	498.73 ± 50.99	**1615.72 ± 176.90**	0.000
B8	*hirustasin_like_Hman01* *hirustasin_like_Hman04* *hirustasin_like_Hman05* *hirustasin_like_Hman06* *hirustasin_like_Hman09*	*hirustasin_like_Wpig1* *hirustasin_like_Wpig2* *hirustasin_like_Wpig3* *hirustasin_like_Wpig4*	975.13 ± 136.89	566.02 ± 59.85	0.192
B9	*hirustasin_like_Hman02* *hirustasin_like_Hman03* *hirustasin_like_Hman07*	*hirustasin_like_Wpig6* *hirustasin_like_Wpig8* *hirustasin_like_Wpig9*	14.32 ± 2.87	64.29 ± 14.27	0.000

TPM, transcripts per million; SE, standard error.

**Table 3:** Comparison of the TPM of representative genes from monophyletic clades with dominantly expressed genes in *H. manillensis* and *W. pigra* (genes with TPM > 1000 are shown in bold).

Branch	Representative Gene		TPM (Mean ± SE)		Mann–Whitney U Test
	*H. manillensis*	*W. pigra*	*H. manillensis*	*W. pigra*	*p*
B1	*hirustasin_like_Hman11*	*guamerin_Wpig*	**2555.10 ± 330.36**	**2237.09 ± 234.68**	0.969
B2	*poecistasin_Hman2*	*—*	**1979.36 ± 323.89**	—	—
B7	*bdellastasin_Hman*	*bdellastasin_Wpig*	498.73 ± 50.99	**1615.72 ± 176.90**	0.000
B8	*hirustasin_like_Hman01*	*hirustasin_like_Wpig1*	**2608.04 ± 476.68**	782.95 ± 128.68	0.001

TPM, transcripts per million; SE, standard error.

**Table 4:** Comparison of the TPM among different geographic populations of *H. manillensis* (genes with TPM > 1000 are shown in bold).

Genes	GXLZ	GDZJ	HNDA	YNHH
*guamerin_Hman*	36.48 ± 21.16	23.99 ± 7.06	**1697.73 ± 373.62**	**2478.44 ± 379.26**
*piguamerin_Hman*	31.60 ± 5.99	42.94 ± 22.19	336.70 ± 130.25	556.96 ± 245.52
*bdellastasin_Hman*	588.99 ± 123.29	285.14 ± 50.86	757.15 ± 105.23	363.63 ± 28.03
*poecistasin_Hman1*	1.16 ± 0.77	0.35 ± 0.35	26.50 ± 8.39	144.45 ± 34.22
*poecistasin_Hman2*	**2027.26 ± 450.90**	**1228.96 ± 239.53**	**4308.24 ± 762.49**	352.98 ± 130.15
*hirustasin_Hman*	18.17 ± 6.32	20.52 ± 17.59	327.05 ± 68.53	5.12 ± 3.82
*hirustasin_like_Hman01*	**3208.87 ± 696.96**	865.97 ± 170.41	**5892.71 ± 1149.24**	464.60 ± 128.39
*hirustasin_like_Hman02*	0.18 ± 0.18	0.00 ± 0.00	0.00 ± 0.00	0.00 ± 0.00
*hirustasin_like_Hman03*	56.58 ± 18.00	34.25 ± 11.71	66.05 ± 11.41	4.73 ± 1.61
*hirustasin_like_Hman04*	**1240.43 ± 312.66**	545.94 ± 158.50	**3535.01 ± 1094.58**	189.93 ± 43.14
*hirustasin_like_Hman05*	908.83 ± 185.79	35.25 ± 14.40	1200.87 ± 359.11	13.75 ± 9.83
*hirustasin_like_Hman06*	289.22 ± 67.17	95.74 ± 16.06	534.75 ± 88.00	480.38 ± 150.49
*hirustasin_like_Hman07*	0.11 ± 0.07	0.64 ± 0.24	0.78 ± 0.29	8.56 ± 2.55
*hirustasin_like_Hman08*	371.95 ± 56.79	141.25 ± 20.37	476.38 ± 68.14	450.94 ± 35.52
*hirustasin_like_Hman09*	0.42 ± 0.26	0.00 ± 0.00	0.00 ± 0.00	0.00 ± 0.00
*hirustasin_like_Hman10*	28.02 ± 7.04	20.86 ± 3.47	108.27 ± 19.24	57.25 ± 3.98
*hirustasin_like_Hman11*	**1169.45 ± 283.54**	786.68 ± 152.10	**4440.28 ± 668.90**	**3824.01 ± 454.00**
*hirustasin_like_Hman12*	0.00 ± 0.00	0.00 ± 0.00	0.00 ± 0.00	0.00 ± 0.00
total	9977.47 ± 1972.10	4128.47 ± 640.15	23,708.45 ± 3815.56	9395.75 ± 1421.22

GXLZ, Liuzhou, Guangxi; GDZJ, Zhanjiang, Guangdong; HNDA, Ding’an, Hainan; YNHH, Honghe, Yunnan.

**Table 5:** Comparison of the TPM among different geographic populations of *W. pigra* (genes with TPM > 1000 are shown in bold).

Genes	HBZX	TJBD	SCYB	HBWH
*guamerin_Wpig*	**1270.04 ± 189.42**	**1664.56 ± 355.67**	**3207.58 ± 354.58**	**2806.17 ± 620.90**
*piguamerin_Wpig*	7.54 ± 2.37	38.67 ± 16.09	98.46 ± 21.39	127.61 ± 84.12
*bdellastasin_Wpig*	**1330.45 ± 321.41**	**1237.76 ± 248.67**	**2540.08 ± 445.48**	**1354.59 ± 226.68**
*hirustasin_Wpig*	285.57 ± 103.50	746.39 ± 191.20	930.92 ± 172.18	938.42 ± 316.45
*hirustasin_like_Wpig1*	325.44 ± 17.90	333.95 ± 94.92	**1632.20 ± 314.95**	840.19 ± 217.86
*hirustasin_like_Wpig2*	36.20 ± 10.60	218.95 ± 68.79	**1133.99 ± 224.77**	591.34 ± 230.18
*hirustasin_like_Wpig3*	71.26 ± 14.58	118.98 ± 36.86	638.90 ± 169.57	325.47 ± 127.52
*hirustasin_like_Wpig4*	121.26 ± 54.36	271.15 ± 85.52	**1550.02 ± 414.79**	847.01 ± 286.88
*hirustasin_like_Wpig5*	253.76 ± 38.68	876.63 ± 156.97	**2000.35 ± 450.54**	**2901.13 ± 650.71**
*hirustasin_like_Wpig6*	81.80 ± 18.92	209.64 ± 72.76	64.51 ± 8.90	369.06 ± 103.62
*hirustasin_like_Wpig7*	6.90 ± 1.70	9.26 ± 2.10	7.51 ± 2.49	24.37 ± 4.92
*hirustasin_like_Wpig8*	2.72 ± 1.28	1.71 ± 0.43	0.14 ± 0.10	0.78 ± 0.39
*hirustasin_like_Wpig9*	14.14 ± 3.48	25.51 ± 4.92	0.64 ± 0.28	0.81 ± 0.66
*total*	3807.07 ± 504.96	5753.17 ± 1201.59	13,805.31 ± 1621.15	11,126.94 ± 2161.89

HBZX, Zhongxiang, Hubei; TJBD, Baodi, Tianjin; SCYB, Yibin, Sichuan; HBWH, Wuhan, Hubei.

## Data Availability

The original contributions presented in this study are included in the article/Supplementary Materials. Further inquiries can be directed to the corresponding authors.

## Supplementary Materials

The following supporting information can be downloaded at: https://www.mdpi.com/article/10.3390/genes16111332/s1, Supplementary Table S1: TPM values of hirustasin gene superfamily members of 40 *Hirudinaria manillensis* samples; Supplementary Table S2: TPM values of hirustasin gene superfamily members of 40 *Whitmania pigra* samples; Supplementary Data S1: Full sequences of hirustasin gene superfamily from *Hirudinaria manillensis* and *Whitmania pigra* used in this study.

## Abbreviations

The following abbreviations are used in this manuscript:

*W. pigra*	*Whitmania pigra* (Whitman, 1884)
*H. manillensis*	*Hirudinaria manillensis* (Lesson, 1842)
TPM	transcripts per million
CVDs	cardiovascular diseases
PPRC	Pharmacopoeia of the People’s Republic of China
TCM	traditional Chinese medicine
CDSs	coding sequences
SE	standard error
GXLZ	Liuzhou, Guangxi
GDZJ	Zhanjiang, Guangdong
HNDA	Ding’an, Hainan
YNHH	Honghe, Yunnan
HBZX	Zhongxiang, Hubei
TJBD	Baodi, Tianjin
SCYB	Yibin, Sichuan
HBWH	Wuhan, Hubei
